# Supplementary material for: Structure of human Cdc45 and implications for CMG helicase function
Source: Nat Commun. 2016 May 18;7:11638. doi: 10.1038/ncomms11638 (PMC4873980; doi:10.1038/ncomms11638)
Supplement: Supplementary Information — Supplementary Figures 1-8. [file ncomms11638-s1.pdf]

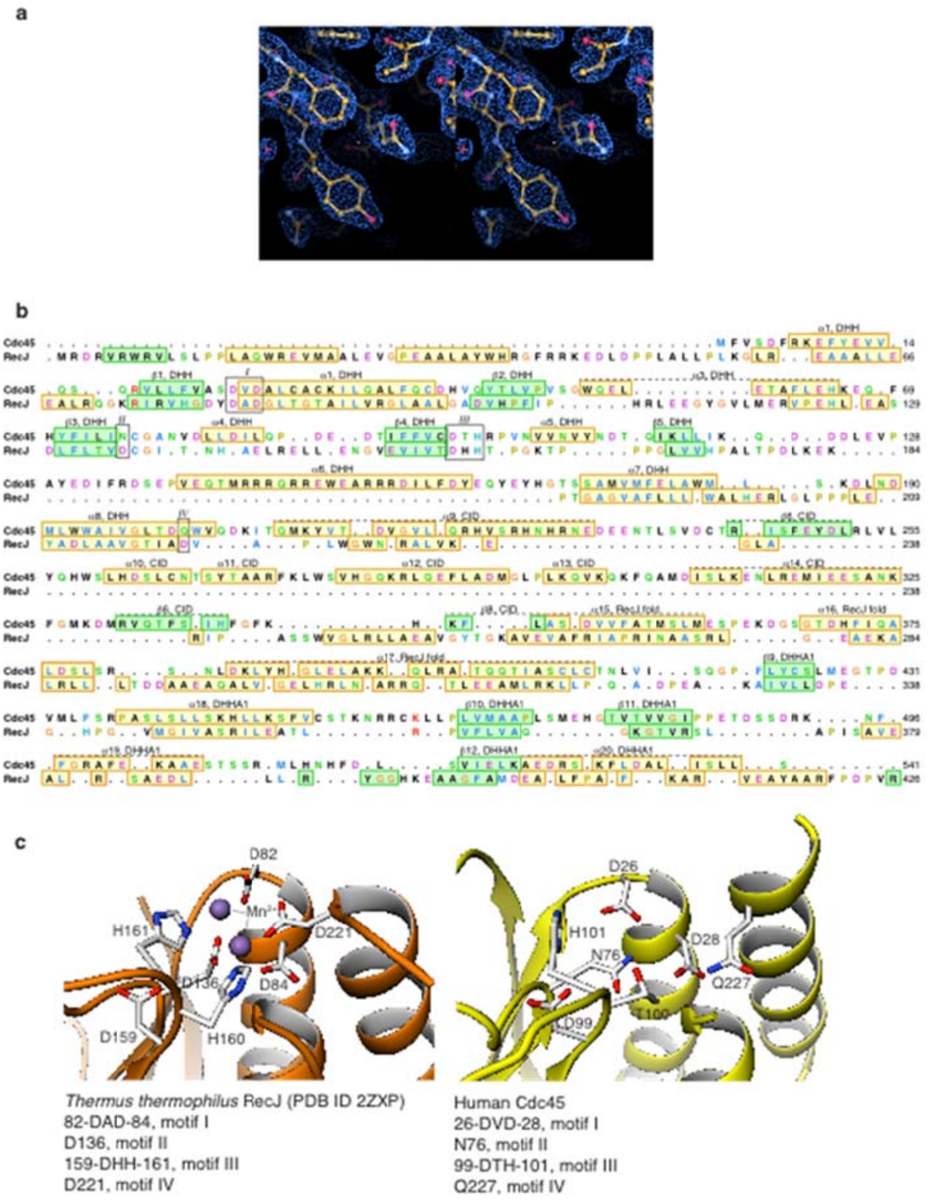


**Supplementary Figure 1** Details of the refined crystallographic model of human Cdc45 and comparison of its active-site region with that of bacterial RecJ. (**a**) Stereo view of a representative example of the final $2F_o$-$F_c$ electron density map, contoured at 1.3 sigmas in Coot, superimposed on the refined Cdc45 model. (**b**) Structure-based amino acid sequence alignment of human Cdc45 and *Thermus thermophilus* RecJ. The alignment is annotated as in Figure 1c. (**c**) Detailed comparison of the active sites of bacterial RecJ (top panel) and human Cdc45 (bottom).

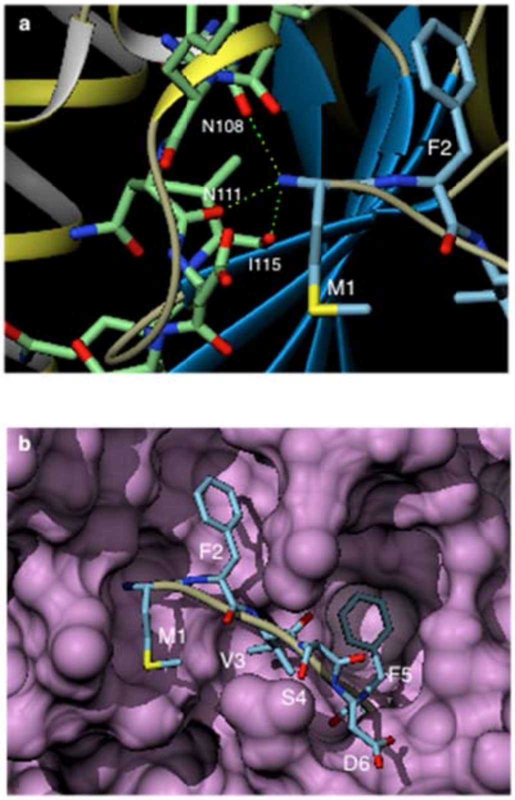


**Supplementary Figure 2** Details of intramolecular interactions at the Cdc45 N-terminus. The top panel emphasises polar contacts, comprising three hydrogen bonds donated by the N-terminal mainchain amino group to the carbonyl moieties of N108, N111 and I115. The bottom panel highlights hydrophobic and aromatic sidechain packing of residues M1, F2, V3 and F5 against the rest of the DHH domain, drawn as a water-accessible molecular surface.

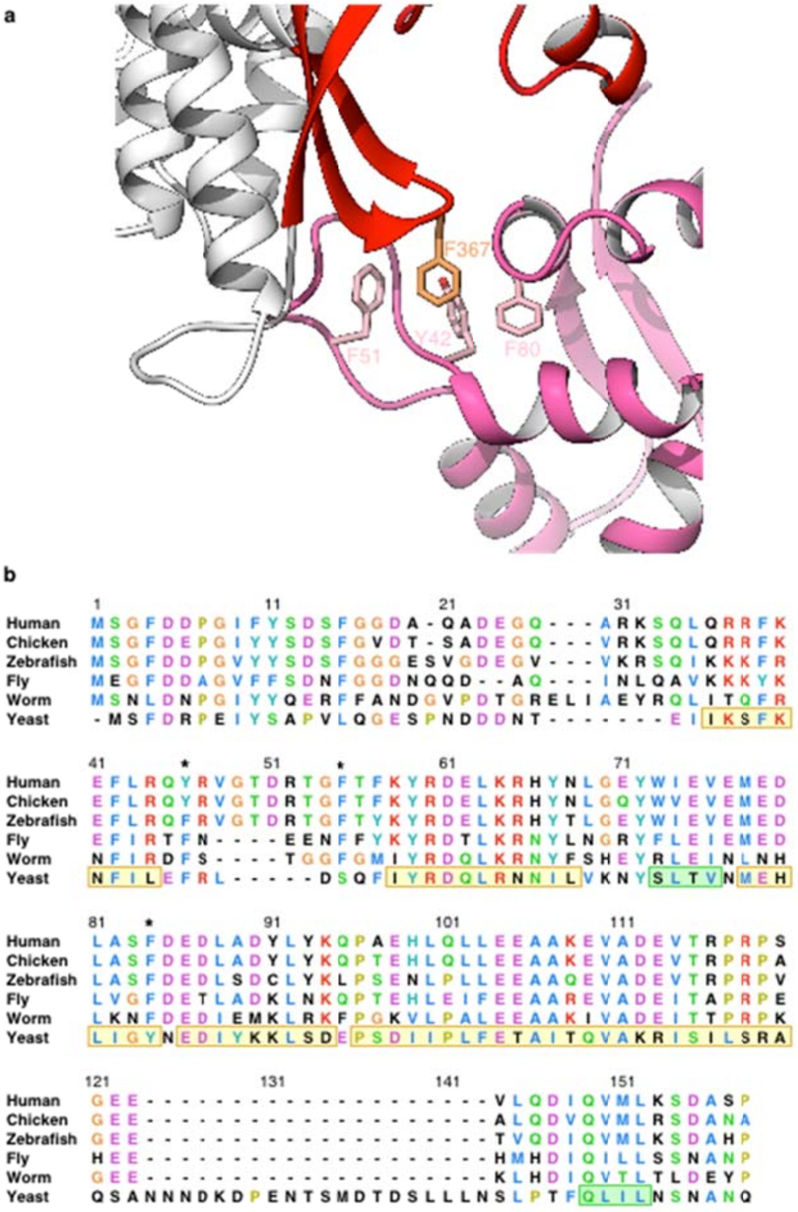


**Supplementary Figure 3** A model of the interaction of Cdc45 (red) with the A-subdomain of MCM5 (pink). Panel (**a**) shows details of the interaction, with the sidechains of human Cdc45's F367 and MCM5's Y42, F51 and F80 shown as sticks. The model for MCM5's A-subdomain structure was built using Phyre2. Panel (**b**) shows a multiple sequence alignment of the A subdomains of MCM5. The secondary structure elements of the yeast MCM5 sequence are shown as yellow (helices) and green (strands) boxes. The position of Y42, F51 and F80 is marked with an asterisk, above the alignment.

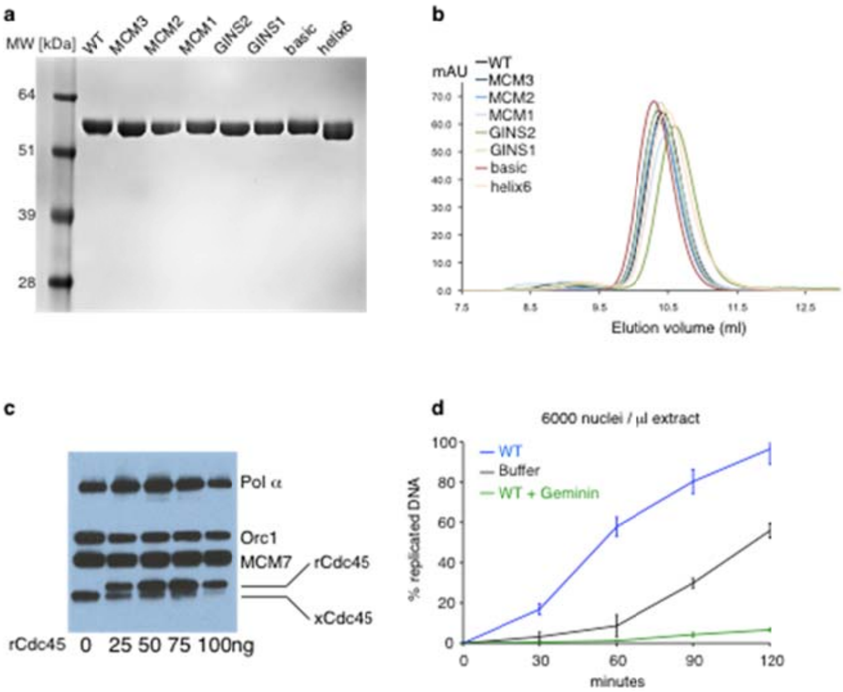


**Supplementary Figure 4** Purification of the *Xenopus* Cdc45 mutants and chromatin recruitment and DNA synthesis assays of wild-type recombinant Cdc45 in *Xenopus* egg extracts. Panels (**a**) and (**b**) show the SDS-PAGE of the purified *Xenopus* Cdc45 mutants and the final gel-filtration step of the purification, respectively. (**c**) Western blot of chromatin recruitment for increasing concentrations of wild-type Cdc45 after 60 min incubation (rCdc45: recombinant Cdc45; xCdc45: endogenous Cdc45). (**d**) DNA synthesis assay in *Xenopus* egg extracts (6000 nuclei/μl), showing higher levels of DNA synthesis relative to the buffer control in extracts containing 100ng/μl wild-type recombinant Cdc45. As expected, DNA synthesis is inhibited by simultaneous addition of the replication inhibitor Geminin.

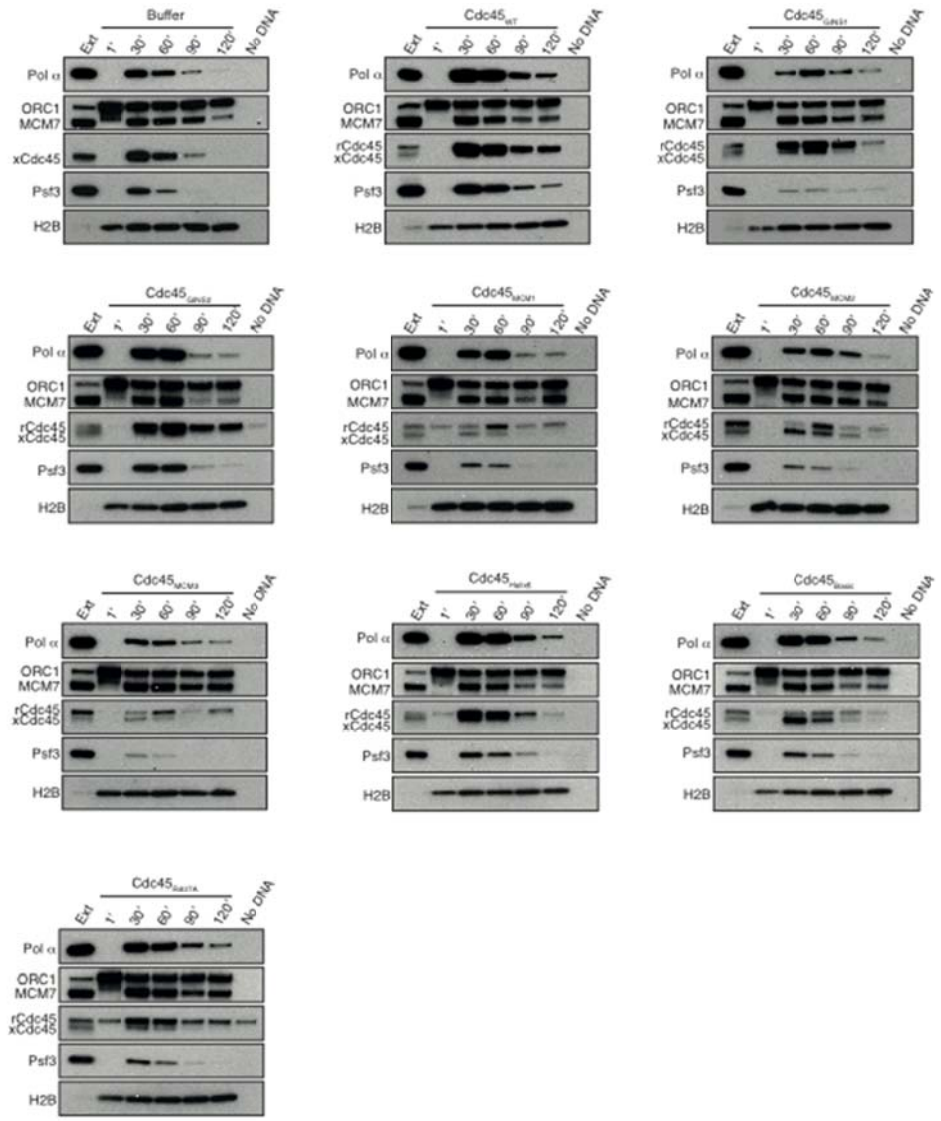


**Supplementary Figure 5** Time-course experiments of chromatin association of *Xenopus* DNA replication factors, after addition of wild-type and mutant recombinant Cdc45 proteins. Each panel shows the Western blot of a time course experiment, after addition of either buffer, wild-type Cdc45 protein or each of the mutant Cdc45 proteins. Extracts were supplemented either with buffer, wild-type Cdc45 protein or the indicated Cdc45 mutant at a final concentration of 100 ng/µl. Immunoblotting was carried out on isolated chromatin fractions after incubation for the indicated time points of 4000 sperm nuclei/µl extracts or the same volume of EB buffer (no DNA). 2.5 ng of each recombinant protein or the same volume of buffer were mixed with 1 µl of interphase extract to be loaded as input lane (Ext). rCdc45: recombinant Cdc45; xCdc45: endogenous Cdc45.

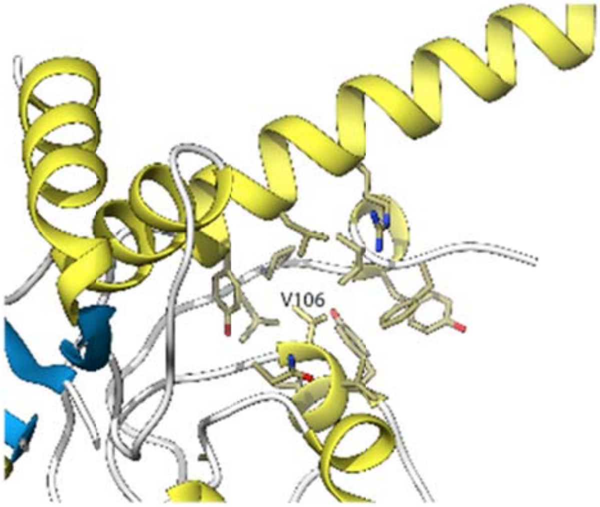


**Supplementary Figure 6** The L131P mutation of the yeast *Cdc45-27* allele, which shows weakened affinity for Sld3 (ref. 53), corresponds to V106 of human Cdc45. The figure shows the position of V106 in the crystal structure of human Cdc45. The structure is drawn as in Fig. 1a; the side chain of V106 and surrounding amino acids are coloured in lighter and darker shades of brown, respectively.

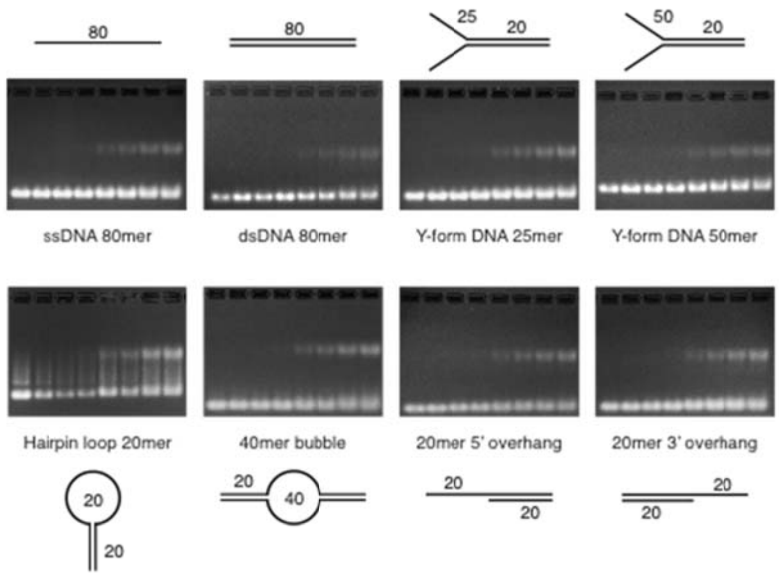


**Supplementary Figure 7** Electrophoretic mobility shift assays (EMSA) for binding of human Cdc45 to a variety of DNA substrates. The type of DNA substrate used in each assay is drawn above each EMSA panel. Details of the experiment are reported in the Methods.

#

#

#

#

#

#

#

#

#

#

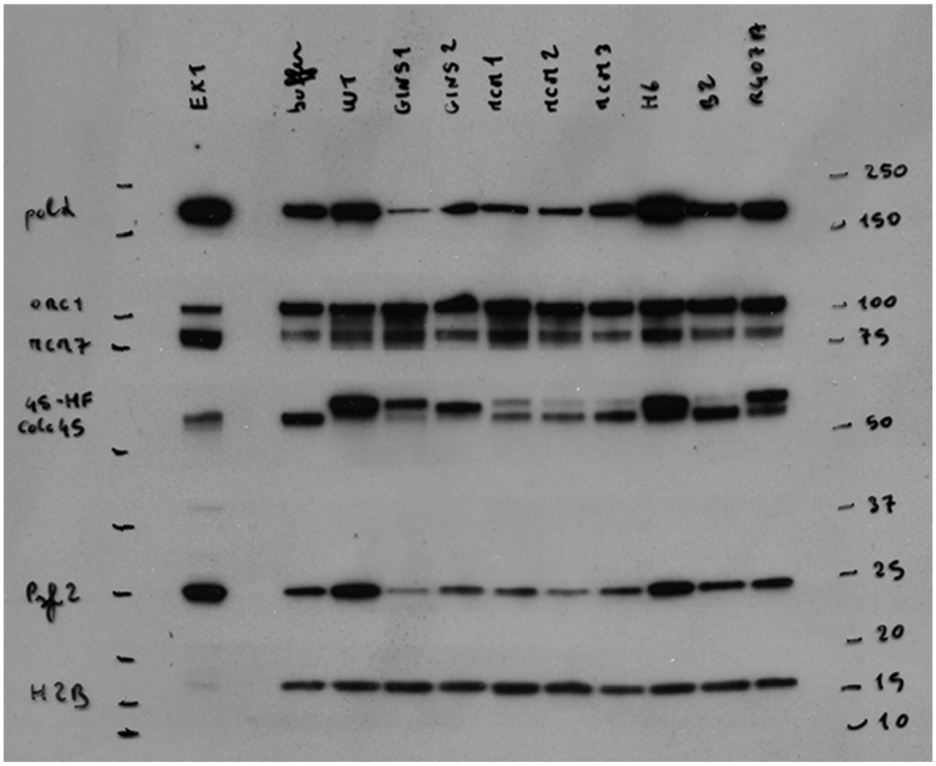


#

**Supplementary figure 8** Uncropped version of the Western blot in Figure 8b.#
